# Population-based study of breast cancer in older women: prognostic factors of relative survival and predictors of treatment

**DOI:** 10.1186/1471-2407-12-472

**Published:** 2012-10-15

**Authors:** Pegdwende Olivia Dialla, Tienhan Sandrine Dabakuyo, Sophie Marilier, Julie Gentil, Patrick Roignot, Ariane Darut-Jouve, Marie-Laure Poillot, Valérie Quipourt, Patrick Arveux

**Affiliations:** 1 Breast and Gynaecologic Cancer Registry of Cote d’Or, Centre Georges François Leclerc, 1 rue Professeur Marion BP 77980, Dijon Cedex, 21079, France; 2 EA 4184, Faculty of Medicine, University of Burgundy, 7 boulevard Jeanne d’Arc, Dijon, 21000, France; 3 Unité Pilote de Coordination en Oncogériatrie en Côte-d’Or, Hôpital de jour Gériatrique, Hôpital de Champmaillot, 2 rue Jules Violle, Dijon Cedex, 21079, France; 4 Centre de Pathologie, 33 rue Nicolas Bornier, Dijon, 21000, France; 5 Clinique Clément-Drevon, 9 rue Princes de Condé, Dijon, 21000, France

**Keywords:** Breast cancer, Elderly women, Predictors of treatment, Prognostic factors, Relative survival

## Abstract

**Background:**

A large proportion of women with breast cancer (BC) are elderly. However, there is a lack of information regarding BC prognostic factors and care in this population. The aims of this study were to assess the prognostic factors of relative survival (RS) among women with BC aged ≥ 75 years old and to identify the predictive factors of treatments administered to this population.

**Methods:**

A population-based study was performed using data from the Cote d’Or breast and gynaecological cancer registry. Women aged 75 years and older with primary invasive BC and resident in Cote d’Or at the time of diagnosis made between January 1998 and December 2008 were retrospectively selected. Prognostic factors of RS were estimated in a generalized linear model with a Poisson error structure. RS rate for the whole population was given at 5 years. Logistic regression models were used to identify the predictors of the treatments administered.

**Results:**

Six hundred and eighty-one women were included. Median age at diagnosis was 80. Comorbidities (p=0.02), pT stage (p=0.04), metastases (p=<0.001), having a family doctor (p=0.03) and hormone-receptor status (p=0.006) were independent prognostic factors of RS. The RS rate at 5 years for the whole population was 78.2%, 95%CI = [72.2-83.0]. Age, pT stage, metastases, histoprognostic SBR grade, hormone receptor status and comorbidities were frequently found to be predictors of treatment with surgery alone, hormone therapy alone, breast conserving surgery plus adjuvant therapy and mastectomy plus adjuvant therapy.

**Conclusions:**

Comorbid conditions adversely affect survival in older women with breast cancer. Moreover the results of this study showed that there are numerous predictors of the type of treatment administered, and that the most important were age and comorbidities.

## Background

Breast cancer (BC) is the most common malignancy in women in France [1-4]. With the aging population and the increase in life expectancy, cancer in elderly people is increasing and this now constitutes a major public health concern.

Many studies have reported data about the prognostic factors of survival among women with BC [5,6]. Despite improved knowledge of these prognostic factors, there are no data about the prognostic factors of relative survival (RS) among older women with BC in a whole population.

The treatment strategy for BC is based on the initial stage of the tumour, the patient’s age, general health, tumour histology and prognostic factors [7]. Progress made in the diagnosis and therapeutic management of BC [8-10] has led to improvements in prognosis and overall quality of survival [11]. However, elderly women with BC are currently undertreated in comparison with the youngest, and this leads to a considerable decrease in specific survival [12-14]. Current therapeutic approaches in older women are most often empiric. Therapeutic decisions are based on wishful thinking on behalf of patients and their loved ones and on the clinician’s impressions [15-18]. Moreover, 12% of patients with BC aged ≥ 80 years old do not receive treatment and 44% do not undergo surgery. Older women are also less likely to receive treatment in compliance with guidelines [19]. In addition, comorbidities, which are particularly common among older women, complicated the management of cancer treatment [20].

Contrary to a long-standing belief, BC can be as aggressive in elderly patients as in younger women. Consequently, the management of older patients with BC should not differ from that in young patients. However, the predictors of treatment in the elderly have not yet been clearly assessed [13,14].

Cancer registries provide a unique opportunity to assess survival and to evaluate the predictors of treatments given in routine practice in this population on comprehensive population-based data.

The first aim of this study was to assess the prognostic factors of RS among women with BC aged ≥ 75 years. The secondary purpose was to identify the predictors of treatment in this population.

## Methods

### Population

A population-based study was undertaken using data from the Cote d’Or breast and gynaecological cancer registry. This registry is the only one in France that focuses on breast and gynaecological cancers. It has been collecting comprehensive population-based data since 1982. Women aged 75 years and older with primary invasive BC and residing in Cote d’Or at the time of diagnosis made between January 1998 and December 2008 were retrospectively selected. Women lost at the date of diagnosis were excluded. For patients with synchronous bilateral BC, the most advanced tumour according to the number of positive nodes, the tumour size, the Scarff, Bloom, and Richardson (SBR) grade, and the tumour histology was included.

### Studied variables and end points

All patients were staged according to the TNM system [21]. We considered the pathologic TNM (pTNM) classification or, when absent, the clinical TNM (cTNM) classification. We used the cTNM for the predictors of treatments because the categories of treatments included surgery or other exclusive treatments without surgery; the pTNM could therefore not be used, and for the prognostic factors we used the pTNM, replaced by the cTNM when the patients were not treated with surgery.

Age at diagnosis was categorized into three classes: 75-79 years, 80-84 years and ≥ 85 years. Pathological tumour size and the number of removed and positive nodes were also recorded. Removed nodes were categorized into two classes: ≤ 10 nodes and > 10 nodes and positive nodes into three classes: 0, 1-3 and ≥ 4 nodes. Tumour characteristics, such as SBR grade classified as grades 1+2 or grade 3, and hormone receptor status, considered positive when oestrogen and/or progesterone were positive, were also collected. Clinical and demographic data, such as the circumstances of diagnosis (clinical diagnosis or individual screening), the matrimonial status (single or not), having a family doctor (yes or no), the vital status (dead or alive) and the place of residence (rural or urban) were recorded too. The comorbidities were collected at the diagnosis, as were all of the diseases associated with BC at the time of diagnosis: diabetes, high blood pressure, neurological disease (Alzheimer, Parkinson, epilepsy), psychiatric illness, tuberculosis, thyroid disorder, genetic abnormalities and previous history of disease affecting treatment administration. Comorbidities were classified in two categories: at least one comorbidity and no comorbidity. The period of diagnosis was split into two: 1998-2003 and 2004-2008 according to the median of distribution. Treatments were grouped into exclusive categories: surgery alone, hormone therapy alone, breast-conserving surgery plus adjuvant therapy (BCS plus adjuvant therapy) and mastectomy plus adjuvant therapy. Patients without treatment constituted the “no treatment” category, and the “others” category was used for the following exclusive treatments: chemotherapy alone, radiotherapy alone, chemotherapy plus hormone therapy, chemotherapy plus radiotherapy, radiotherapy plus hormone therapy, chemotherapy plus radiotherapy plus hormone therapy, neoadjuvant chemotherapy plus surgery plus adjuvant therapy and neoadjuvant chemotherapy plus surgery.

Survival was calculated from the date of diagnosis until the date of death or the date of last follow-up. The cut-off date for the survival analysis was set at 01 January 2010. Patients who were alive after the cut-off date were censored.

### Statistical methods

Quantitative variables are given as means, standard deviations (SD), medians and ranges, while qualitative variables are given as percentages. The percentage of missing values is also provided.

Treatments categories were compared according to the follow-up periods using Chi2 tests. Cochran-Armitage trend tests were used to compare treatments according to the age classes.

RS rate with the 95% confidence interval (CI) for the whole population was given at 5 years. RS is an estimator of the excess mortality ratio (EMR) estimated from life tables as the ratio of the observed survival of the patients (where all deaths are considered events) to the expected survival (ES) [22]. The ES was estimated using Cote d’Or female life-expectancy tables stratified by age and period-matched mortality rates. The EMR was estimated in a generalized linear model with a Poisson error structure [23]. Follow-up time was stratified in annual intervals. Prognostic factors of survival were assessed using univariate and multivariate analyses of RS.

Univariate and multivariate logistic regression models were performed to identify the predictive factors of the administration of the different type of exclusive treatments: surgery alone, hormone therapy alone, BCS plus adjuvant therapy alone and mastectomy plus adjuvant therapy alone. Odds Ratios (OR) and their 95% confidence intervals (CI) are given.

For RS and logistic regression, all variables with a univariate p*-*value ≤ 0.20 were eligible for multivariate analyses. Correlations and interactions were tested for eligible variables. To prevent co-linearity, when two variables were significantly correlated, one variable was retained according to its clinical relevance or to the value of the likelihood ratio. All reported p values are two sided. The statistical significance level was set at p<0.05. Analyses were done using SAS (Statistical Analysis system version 9.2) and or STATA (version 9.0).

### Ethics statement

The Breast and Gynaecologic Cancer Registry of Cote d’Or was approved by the CNIL (National Commission on Informatics and Liberties) for the collection and recording data for research purposes (authorization number DR-2012-038).

## Results

### Patients’ characteristics

Seven hundred and fifteen (715) women aged 75 years and older with invasive primary BC were registered from January 1998 to December 2008. Among them, 34 were lost to follow-up at the date of diagnosis. Finally, 681 patients were included in the study. The median age at diagnosis was 80 (range, 75 to 98 years). The clinical and pathological features of the studied population are summarized in Table 1.

**Table 1:** Characteristics of patients (N=681) and tumours

**Variables**	**N (681)**	**%**	**Mean (SD)**	**Median [min;max]**
Age at diagnosis (years)	681		81.3 (5.2)	80.0 [75;98]
Pathological tumor size (mm)	546		27.0 (19.8)	22.0 [2;160]
Nodes removed	612		8.2 (6.3)	8.0 [0;32]
Positive nodes	519		2.0 (4.0)	0.0 [0;32]
Age				
75-79	310	45.5		
80-84	208	30.5		
≥ 85	163	23.9		
Unknown	0	0.0		
T stage				
T0	8	1.2		
T1	214	31.4		
T2	186	27.3		
T3	19	2.8		
T4	108	15.9		
Unknown	146	21.4		
N stage				
N0	451	66.2		
N1	112	16.4		
N2	10	1.5		
N3	2	0.3		
Unknown	106	15.6		
M stage				
M0	579	85.0		
M1	64	9.4		
Unknown	38	5.6		
pT stage				
p T1	259	38.0		
p T2	206	30.2		
p T3	20	2.9		
p T4	64	9.4		
p T*	130	19.1		
Unknown	2	0.3		
pN stage				
p N0	288	42.3		
p N1	227	33.3		
pN*	129	18.9		
Unknown	37	5.4		
pM stage				
p M0	576	84.6		
p M1	64	9.4		
Unknown	41	6.0		
pT stage†				
pT0	1	0.1		
p T1	282	41.4		
p T2	245	36.0		
p T3	26	3.8		
p T4	104	15.3		
Unknown	23	3.4		
pN stage†				
p N0	350	51.4		
p N+	269	39.5		
Unknown	62	9.1		
pM stage†				
p M0	576	84.6		
p M1	64	9.4		
Unknown	41	6.0		
Nodes removed				
Nodes ≤10	389	57.1		
Nodes > 10	223	32.7		
Unknown	69	10.1		
Positive nodes				
0	290	42.6		
1 - 3	136	20.0		
≥ 4	93	13.7		
Unknown	162	23.8		
Histoprognostic SBR grade				
1	132	19.4		
2	350	51.4		
3	160	23.5		
Unknown	39	5.7		
Diagnosis circumstances				
Clinic	471	69.2		
Individual screening	109	16.0		
Unknown	101	14.8		
Place of residence				
Urban	474	69.6		
Rural	203	29.8		
Unknown	4	0.6		
Hormone receptors				
Positive	590	86.6		
Negative	80	11.7		
Unknown	11	1.6		
Comorbidities				
Yes	419	61.5		
No	163	23.9		
Unknown	99	14.5		
Period				
1998-2003	321	47.1		
2004-2008	360	52.9		
Unknown	0	0.0		
Matrimonial status				
Not single	183	26.9		
Single‡	288	42.3		
Unknown	210	30.8		
Having a family doctor				
Yes	621	91.2		
No	51	7.5		
Unknown	9	1.3		
Vital status				
Dead	305	44.8		
Alive	376	55.2		
Unknown	0	0.0		

Abbreviations: N = number of patients; % = percentage; SD = standard deviations. ^*^Patients without surgery;

†For patients without surgery pTNM stage was replaced by TNM; ^‡^Patient single, divorced or widowed.

Information on comorbidities was missing for 14.5% of the women. The comorbidities recorded were: high blood pressure (27%), diabetes (5.4%), neurological disease (3%), obesity (1.2%), psychiatric illness (1%), tuberculosis (1.5%), thyroid disorder (4%), previous history of stroke, heart failure or thrombosis (8%), and previous history of disease affecting treatment administration (10%).

### Treatment description

Concerning treatment, 52 (7.6%) underwent surgery alone, and hormone therapy alone was given to 74 (10.9%). BCS plus adjuvant therapy was given to 238 (34.9) while 211 patients (31%) received mastectomy plus adjuvant therapy. Nineteen (2.8%) did not receive treatment and 26 patients (3.8%) were treated by other treatments: chemotherapy alone or radiotherapy alone or chemotherapy plus hormone therapy or chemotherapy plus radiotherapy or radiotherapy plus hormone therapy or chemotherapy plus radiotherapy plus hormone therapy or neoadjuvant chemotherapy plus surgery plus adjuvant therapy or neoadjuvant chemotherapy plus surgery. For 1998-2003 and 2004-2008, the use of BCS plus adjuvant therapy (p=0.63), mastectomy plus adjuvant therapy (p=0.52) and other treatments (p=0.43) was similar. In contrast, patients were less often treated by surgery alone in the second period (p<0.001): 11.2% and 4.4% for 1998-2003 and 2004-2008, respectively. The use of hormone therapy alone was more frequent in the second period (p=0.001): 6.5% and 14.7%, respectively (Table 2).

**Table 2:** Treatment description by period

**Period**	**1998-2003 (N= 321)**		**2004-2008 (N=360)**		p value*	**All cases (N=681)**	
**Treatments**	**321**	**%**	**360**	**%**		**681**	**%**
No treatment					0.65		
Yes	10	3.1	9	2.5		19	2.8
No	306	95.3	340	94.4		646	94.9
Unknown	5	1.6	11	3.1		16	2.3
Surgery alone					<0.001		
Yes	36	11.2	16	4.4		52	7.6
No	254	79.1	328	91.1		582	85.5
Unknown	31	9.7	16	4.4		47	6.9
Hormone therapy					0.001		
Yes	21	6.5	53	14.7		74	10.9
No	264	82.2	282	78.3		546	80.2
Unknown	36	11.2	25	6.9		61	9.0
Breast conserving surgery plus adjuvant therapy					0.63		
Yes	113	35.2	125	34.7		238	34.9
No	193	60.1	231	64.2		424	62.3
Unknown	15	4.7	4	1.1		19	2.8
Mastectomy plus adjuvant therapy					0.52		
Yes	95	29.6	116	32.2		211	31.0
No	210	65.4	230	63.9		440	64.6
Unknown	16	5.0	14	3.9		30	4.4
Others†					0.43		
Yes	10	3.1	16	4.4		26	3.8
No	275	85.7	319	88.6		594	87.2
Unknown	36	11.2	25	6.9		61	9.0

Abbreviations: N = number of patients.

*Chi 2 test.

†Others: chemotherapy alone, radiotherapy alone, chemotherapy plus hormone therapy, chemotherapy plus radiotherapy, radiotherapy plus hormone therapy, chemotherapy plus radiotherapy plus hormone therapy, neoadjuvant chemotherapy plus surgery plus adjuvant therapy, neoadjuvant chemotherapy plus surgery.

Older patients were more likely to be treated with surgery alone (p=0.02) and with hormone therapy alone (p<0.001) and were more likely to receive no treatment (p=0.006). BCS plus adjuvant therapy (p<0.001) and other treatments (p=0.03) were less likely to be given to older patients. In contrast, the use of mastectomy plus adjuvant therapy did not vary with age (p=0.40) (Table 3).

**Table 3:** Treatment description by age

**Age**	**75-79 (N=310)**		**80-84 (N=208)**		**≥85 (N=163)**		p value*	**All cases (N=681)**	
**Treatments**	**310**	**%**	**208**	**%**	**163**	**%**		**681**	**%**
No treatment							0.006		
Yes	4	1.3	6	2.9	9	5.5		19	2.8
No	304	98.1	197	94.7	145	89.0		646	94.9
Unknown	2	0.6	5	2.4	9	5.5		16	2.3
Surgery alone							0.02		
Yes	19	6.1	13	6.3	20	12.3		52	7.6
No	272	87.7	184	88.5	126	77.3		582	85.5
Unknown	19	6.1	11	5.3	17	10.4		634	93.1
Hormone therapy							<0.001		
Yes	12	3.9	18	8.7	44	27.0		74	10.9
No	277	89.4	176	84.6	93	57.1		546	80.2
Unknown	21	6.8	14	6.7	26	16.0		61	9.0
Breast conserving surgery plus adjuvant therapy							<0.001		
Yes	144	46.5	69	33.2	25	15.3		238	34.9
No	157	50.6	135	64.9	132	81.0		424	62.3
Unknown	9	2.9	4	1.9	6	3.7		19	2.8
Mastectomy plus adjuvant therapy							0.40		
Yes	92	29.7	83	39.9	36	22.1		211	31.0
No	208	67.1	116	55.8	116	71.2		440	64.6
Unknown	10	3.2	9	4.3	11	6.7		30	4.4
Others†							0.03		
Yes	18	5.8	5	2.4	3	1.8		26	3.8
No	271	87.4	189	90.9	134	82.2		594	87.2
Unknown	21	6.8	14	6.7	26	16.0		61	9.0
									

Abbreviations: N = number of patients.

*Cochran-Armitage trend test.

†Others: chemotherapy alone, radiotherapy alone, chemotherapy plus hormone therapy, chemotherapy plus radiotherapy, radiotherapy plus hormone therapy, chemotherapy plus radiotherapy plus hormone therapy, neoadjuvant chemotherapy plus surgery plus adjuvant therapy, neoadjuvant chemotherapy plus surgery.

### Prognostic factors and analyses of RS

The median follow-up was 3 years (min-max) = (0.01-11.96). At the cut-off, 305 deaths (45%) had occurred. RS rates decreased with follow-up. RS at 1 year for the whole population was 93.5%, 95% CI = [90.5-95.5] and at 5 years was 78.2%, 95% CI = [72.2-83.0].

In the multivariate analysis, the pathological tumor size correlated with the pT stage, and we therefore retained the pT stage for the analyses. Examined and positive nodes correlated with clinical N stage and pN stage, respectively, and we retained the clinical N stage and pN stage for the analyses.

Multivariate RS analyses revealed an increased risk of death in patients with an advanced pT stage: Relative Excess Rates (RER) =2.58, 95% CI = [1.23-5.42], in patients with metastasis at the time of diagnosis: RER=6.86, 95% CI = [3.43-13.73] and in patients with comorbidities: RER=2.35, 95% CI = [1.03-5.36]. In contrast, a decreased risk of death was observed in patients who had a family doctor: RER=0.22, 95% CI = [0.07-0.68] and in patients with hormone-receptor positive tumours: RER=0.33, 95% CI = [0.16-0.70] (Table 4).

**Table 4:** Prognostic factors for relative survival

**Factors**	**N=(681)**	**Number of deaths**	**Univariate analysis**			**Multivariate analysis (N=495)**		
			**RER**	**95% CI**	**p value**	**RER**	**95% CI**	**p value**
Age	681				0.001			0.18
75-79	310	107	1			1		
80-84	208	88	0.99	0.52-1.88		1.01	0.47-2.16	
≥85	163	110	2.75	1.68-4.48		2.15	1.02-4.55	
pT stage	658				<0.001			0.04
pT0+pT1	283	91	0.53	0.24-1.17		1.77	0.72-4.36	
pT2	245	109	1			1		
Pt3+Pt4	130	89	3.89	2.20-6.82		2.58	1.23-5.42	
pN stage	619				<0.001			0.14
pN0	350	132	1			1		
pN+	269	135	3.13	1.68-5.75		1.75	0.80-3.83	
pM stage	640				<0.001			<0.001
pM0	576	227	1			1		
pM1	64	47	10.18	6.11-16.95		6.86	3.43-13.73	
Having a family doctor	672				<0.001			0.03
No	51	41	1			1		
Yes	621	258	0.17	0.11-0.28		0.22	0.07-0.68	
Histoprognostic SBR grade	642				<0.001			0.08
1+2	482	185	1			1		
3	160	93	2.73	1.66-4.51		1.87	0.93-3.79	
Hormone receptors	670				<0.001			0.006
Negative	80	46	1			1		
Positive	590	255	0.38	0.23-0.63		0.33	0.16-0.70	
Comorbidities	582				0.03			0.02
No	163	51	1			1		
Yes	419	179	4.26	1.13-16.28		2.35	1.03-5.36	
Diagnosis circumstances	580				0.10			0.13
Clinic	471	209	1			1		
Individual screening	109	27	0.13	0.01-1.48		0.42	0.11-1.65	
Place of residence	677				0.43			
Rural	203	92	1					
Urban	474	211	0.82	0.50-1.35				
Period	681				0.79			
1998-2003	321	190	1					
2004-2008	360	115	1.07	0.66-1.73				
Matrimonial status	471				0.89			
Single*	288	131	1					
Not single	183	73	0.96	0.53-1.75				

Abbreviations: RER = relative excess rates; CI = confidence interval, N = number of patients.

^*^ Patient single, divorced or widowed.

### Predictive factors of treatments administered

Predictors of the treatments are listed in Table 5. Patients with hormone-receptor positive tumours or patients diagnosed in the period from 2004 to 2008 were less likely to undergo surgery alone. OR were 0.29, 95% CI = [0.12-0.72], and 0.40, 95% CI = [0.20-0.78], respectively. Older patients with metastasis did not receive surgery alone. The oldest patients (≥85 years), patients with metastasis or with comorbidities were more often treated with hormone therapy alone. The oldest patients (80-84 and ≥85 years) or patients with advanced tumours (stages T2 and T3+T4) were less likely to receive BCS plus adjuvant therapy. In contrast, patients with advanced tumours (stages T2 and T3+T4) were more likely to be treated with mastectomy plus adjuvant therapy, OR were 5.57, 95% CI = [3.09-10.03] and 3.60, 95%CI = [1.78-7.29], respectively. Patients with histoprognostic SBR grade 3 were more likely to be treated with mastectomy plus adjuvant therapy, OR= 2.18, 95% CI = [1.26-3.76]. Moreover, there were interactions between age and the circumstances of diagnosis for the predictors of mastectomy plus adjuvant therapy (p<0.001). Indeed, patients aged ≥ 85 years with a clinical diagnosis were 74% less likely to be treated with mastectomy plus adjuvant therapy than were those aged between 75 and 79 years old with a clinical diagnosis.

**Table 5:** Multivariate logistic regression analysis of predictive factors of treatments administered

**Variables**	**OR**	**95% CI**	**p value**
Predictive factors for surgery alone * (N=537)			
Age			0.06
75-79	1		
80-84	1.28	0.57-2.88	
≥85	2.52	1.15-5.54	
N stage			0.05
N0	1		
N+	0.36	0.13-1.01	
Hormone receptors			0.008
Negative	1		
Positive	0.29	0.12-0.72	
Period			0.008
1998-2003	1		
2004-2008	0.40	0.20-0.78	
Histoprognostic SBR grade			0.25
1+2	1		
3	1.59	0.72-3.49	
Place of residence			0.16
Rural	1		
Urban	1.84	0.78-4.33	
**Predictive factors for hormone therapy alone (N=452)**			
Age			<0.001
75-79	1		
80-84	2.07	0.80-5.35	
≥85	16.22	6.74-39.03	
T stage			
T0+T1	1		0.89
T2	1.21	0.53-2.74	
T3+T4	1.22	0.42-3.52	
N stage			0.52
N0	1		
N+	1.32	0.57-3.07	
M stage			0.02
M0	1		
M1	3.67	1.25-10.74	
Comorbidities			<0.001
No	1		
Yes	7.59	2.45-23.56	
Histoprognostic SBR grade			0.22
1+2	1		
3	0.55	0.21-1.43	
Period			0.23
1998-2003	1		
2004-2008	1.56	0.75-3.24	
Hormone receptors			0.07
Negative	1		
Positive	4.73	0.90-24.94	
Diagnosis circumstances			0.55
Clinic	1		
Individual screening	0.72	0.24-2.13	
Place of residence			1
Rural	1		
Urban	1	0.47-2.12	
**Predictive factors for BCS plus adjuvant therapy † (N=381)**			
Age			<0.001
75-79	1		
80-84	0.53	0.30-0.95	
≥85	0.21	0.09-0.50	
T stage			<0.001
T0 +T1	1		
T2	0.17	0.09-0.31	
T3+T4	0.13	0.05-0.30	
N stage			0.23
N0	1		
N+	0.64	0.31-1.33	
Comorbidities			0.11
No	1		
Yes	0.62	0.34-1.12	
Diagnosis circumstances			0.10
Clinic	1		
Individual screening	1.71	0.90-3.24	
Histoprognostic SBR grade			0.30
1+2	1		
3	0.7	0.36-1.37	
Matrimonial status			0.40
Single‡	1		
Not single	1.26	0.73-2.17	
Hormone receptors			0.78
Negative	1		
Positive	1.14	0.46-2.83	
**Predictive factors for mastectomy plus adjuvant therapy (N=381)**			
T stage			<0.001
T0+T1	1		
T2	5.57	3.09-10.03	
T3+T4	3.60	1.78-7.29	
N stage			0.61
N0	1		
N+	0.86	0.49-1.53	
M stage			0.07
M0	1		
M1	0.47	0.21-1.08	
Histoprognostic SBR grade			0.005
1+2	1		
3	2.18	1.26-3.76	
Matrimonial status			0.19
Single‡	1		
Not single	0.72	0.44-1.18	
Place of residence			0.22
Rural	1		
Urban	0.73	0.44-1.20	
Age x diagnosis circumstances §			<0.001
75-79 x clinical diagnosis	1		
80-84 x clinical diagnosis	1.63	0.91-2.93	
≥85 x clinical diagnosis	0.26	0.13-0.52	
75-79 x individual screening	0.45	0.17-1.18	
80-84 x individual screening	0.33	0.09-1.18	
≥85 x individual screening	1.25	0.24-6.38	

Abbreviations: OR = Odds Ratios; CI = confidence interval, N = number of patients.

*Predictive factors for surgery alone without the variable M stage because no patient with stage M1 had surgery alone.

†Predictive factors for BCS plus adjuvant therapy without the variable M stage because only 4 patients with stage M1 received BCS plus adjuvant therapy.

‡patient single, divorced or widowed.

§Interaction age and diagnosis circumstances.

**NB**: Multivariate logistic regression analyses were adjusted for age, TNM stage, period, diagnosis circumstances, comorbidities, histoprognostic SBR grade, tumour type, hormone receptors, matrimonial status, and place of residence.

## Discussion

The results of this study showed that tumours were discovered at an advanced stage in elderly patients. Nine per cent of patients had metastatic tumours and 9.4% had inflammatory tumours or tumours with extension to the skin or to the chest wall.

According to the follow-up period, older patients were less likely to be treated with surgery alone and more likely to receive hormone therapy alone in the more recent period. These results are in line with a study conducted by Bastiaannet et al, which showed that patients aged ≥ 75 years were less likely to undergo surgery and more likely to have hormone therapy [24]. One possible explanation could be that more recently surgery alone was not proposed to treat BC because with the improvement in breast cancer management other, more effective treatments like hormone therapy or treatment combinations have been preferred to surgery alone to treat older women with BC. Other explanations could be that elderly women with BC are too frail and have more advanced tumours associated with the presence of comorbidities.

Regarding the assessment of RS, the analyses were done on comprehensive data. Moreover, in this population-based study, the follow-up for vital status was complete for all included patients with no patients lost to follow-up at the cut-off date. Our multivariate analyses of RS showed that pT stage, metastasis, having a family doctor, hormone receptor status and comorbidities were independent predictors of the length of survival. pT stage, metastasis and hormone receptor status have already been shown to be prognostic factors of survival in BC patients [6]. Indeed, the influence of hormone receptors could be attributed to the efficacy of hormone therapy given to patients with hormone-receptor positive tumours. A recent study showed that comorbidities had a significant impact on survival after BC with poorer survival among old patients with one or more comorbid conditions [25]. Patients who had a family doctor had a lower risk of death than those without a family doctor. This could be explained by the fact that patients who had a family doctor were more likely to be diagnosed with less advanced BC.

Five-year RS for our population was 78.2%. The Eurocare-4 study, which analysed survival in cancer patients diagnosed from 1995 to 1999, showed that 5-year RS in France was 82.7% [26]. Survival was shorter in our population than in the whole population of BC patients in France. This could be due to the characteristics of our population, which was probably older than the population studied in Eurocare 4, and had comorbidities.

The results of this study showed that age was the major predictor of the type of treatment administered. The proportion of patients who had no treatment increased with age. Moreover, except for surgery and mastectomy plus adjuvant therapy, upon which age had no influence, the oldest patients were more often treated with hormone therapy and less likely to be treated with BCS plus adjuvant therapy, probably because of advanced stage of the tumour at discovery and the presence of comorbidities that preclude treatment with BCS in the elderly. These results are in line with those reported in other studies [27,28]. Adjuvant therapy (radiotherapy) after primary surgery was less frequent in the oldest age group with BC. This was not the case for hormone therapy, which was more likely to be administered in the oldest patients than in patients in the youngest age group.

According to the characteristics of the tumour, older patients with an advanced tumour (stage T2, T3+T4) were more likely to undergo mastectomy plus adjuvant therapy and less likely to have BCS plus adjuvant therapy. This could be due to large tumour size which precludes BCS. Treatment with mastectomy plus adjuvant therapy limits organ invasion and thus decreases the risk of disease recurrence in the breast or distant metastasis. Another explanation could be the choice of the physician to avoid adjuvant radiotherapy for extremely frail women. Nevertheless, patients with hormone-receptor positive tumours were less often treated with surgery alone because these patients were usually treated with hormone therapy in accordance with the guidelines of the French health authorities [29]. Patients with metastasis were also more likely to receive hormone therapy, but less often treated with surgery. Surgery is a local treatment and is thus inadequate for the treatment of metastatic disease. Patients with comorbidities were more often treated with hormone therapy. Older patients with comorbidity received less aggressive treatments as shown in another study [28]. Treatments are often modified according to age-related issues such as comorbidity and the general state of health. This highlights the importance of considering comorbidities in the management of BC.

The strength of this study is that the analyses were done in a heterogeneous and exhaustive group of patients, using data from the Cote d’Or breast and gynaecological cancer registry. Therefore, the results could be considered representative of elderly patients living in the department during this period. Cancer registries provide the unique opportunity to evaluate predictors of treatments given in routine practice and to assess survival using comprehensive population-based data. The use of RS makes it possible to correct for non-BC-related deaths while circumventing the problems associated with establishing the cause of death.

## Conclusions

Comorbidity was associated with decreased survival in older women with breast cancer. Moreover, the results of this study showed that there are numerous predictors of the type of treatment administered, of which the most important were age and comorbidities.

## Abbreviations

BC: Breast cancer; BCS: Breast conserving surgery; CI: Confidence intervals; EMR: Excess mortality ratio; ES: Expected survival; RER: Relative excess rates; OR: Odds ratios; RS: Relative survival; SAS: Statistical analysis system; SBR: Scarff bloom and richardson; SD: Standard deviation.

## Competing interests

The authors declare that they have no competing interests.

## Authors’ contributions

Authors’ contributions were as follows: OD, TSD , SM, ,VQ, PA conceived the idea of the study, designed the study, collected data, performed statistical analyses, interpreted data, revised the manuscript and gave final approval to the version to be published. M-LP collected data, revised the manuscript and gave final approval to the version to be published. JG, PR, AD-J revised the manuscript and gave final approval to the version to be published. All authors read and approved the final manuscript.

## Pre-publication history

The pre-publication history for this paper can be accessed here:

http://www.biomedcentral.com/1471-2407/12/472/prepub

## References

[B1] Boyle P Ferlay J Cancer incidence and mortality in Europe 2004 Ann Oncol 2005 16 481 488 10.1093/annonc/mdi098 15718248

[B2] Colonna M Danzon A De Lafosse P Mitton N Bara S Bouvier A-M Ganry O Guizard A-V Launoy G Molinie F Sauleau E-A Schvartz C Velten M Grosclaude P Tretarre B Cancer prevalence in France: time trend, situation in 2002 and extrapolation to 2012 Eur J Cancer 2008 44 115 122 10.1016/j.ejca.2007.10.022 18032021

[B3] Belot A Grosclaude P Bossard N Jougla E Benhamou E Delafosse P Guizard AV Molinié F Danzon A Bara S Bouvier AM Trétarre B Binder-Foucard F Colonna M Daubisse L Hédelin G Launoy G Le Stang N Maynadié M Monnereau A Troussard X Faivre J Collignon A Janoray I Arveux P Buemi A Raverdy N Schvartz C Bovet M Chérié-Challine L Estève J Remontet L Velten M Cancer incidence and mortality in France over the period 1980-2005 Rev Epidemiol Sante Publique 2008 56 159 175 10.1016/j.respe.2008.03.117 18547762

[B4] La situation du cancer en France en 2011 Collection rapports et synthèses, ouvrage collectif Edité par l’INCa 2011 Boulogne-Billancourt

[B5] Kim KJ Huh SJ Yang JH Park W Nam SJ Kim JH Lee JH Kang SS Lee JE Kang MK Park YJ Nam HR Treatment results and prognostic factors of early breast cancer treated with a breast conserving operation and radiotherapy Jpn J Clin Oncol 2005 35 126 133 10.1093/jjco/hyi039 15741302

[B6] Dabakuyo TS Bonnetain F Roignot P Poillot M-L Chaplain G Altwegg T Hedelin G Arveux P Population-based study of breast cancer survival in Cote d'Or (France): prognostic factors and relative survival Ann Oncol 2008 19 276 283 10.1093/annonc/mdm491 17962200

[B7] Andrieu JM Colonna P Cancers: évaluation, traitement et surveillance 1997 Ed ESTEM, Paris

[B8] DiGiovanna MP DeVita VT Jr, Hellman S, Rosenberg SA Clinical significance of HER-2/neu overexpression: Part I Principles and practice of oncology. volume 13 1999 9 Lippincott Williams & Wilkins Cedar Knolls, NJ

[B9] Huiart L Bardou V-J Puig B Maraninchi D Improvement in breast cancer survival between 1975 and 2003 in a cohort of 5722 women Bull Cancer 2006 93 391 399 16627242

[B10] Goldhirsch A Ingle JN Gelber RD Coates AS Thürlimann B Senn H-J Panel members Thresholds for therapies: highlights of the St Gallen intenational expert consensus on the primary therapy of early breast cancer 2009 Ann Oncol 2009 20 1319 1329 10.1093/annonc/mdp322 19535820 PMC2720818

[B11] Maraninchi D Cerf N Bousquet P Lequellec-Nathan M Danzon A Belot A Thuret A Jougla E Rey G Grosclaude P Colonna M Bossard N Canet C Vongmany N Dynamique d’évolution des taux de mortalité des principaux cancers en France 2010 INCa p42 http://www.e-cancer.fr/component/docman/doc_download/5986-dynamique-devolution-des-taux-de-mortalite-des-principaux-cancers-en-france-novembre-2010

[B12] Grosclaude P Colonna M Hedelin G Tretarre B Arveux P Lesec’h JM Raverdy N Sauvage-Machelard M Survival of women with breast cancer in France: variation with age, stage and treatment Breast Cancer Res Treat 2001 70 137 143 10.1023/A:1012974728007 11768604

[B13] Bouchardy C Rapiti E Fioretta G Laissue P Neyroud-Caspar I Schäfer P Kurtz J Sappino A-P Vlastos G Undertreatment strongly decreases prognosis of breast cancer in elderly women J Clin Oncol 2003 21 3580 3587 10.1200/JCO.2003.02.046 12913099

[B14] Van Leeuwen BL Rosenkranz KM Lei Feng L Bedrosian I Hartmann K Hunt KK Kuerer HM Ross M Singletary SE Babiera GV the Department of Surgical Oncology, MD Anderson Cancer Center The effect of under-treatment of breast cancer in women 80 years of age and older Crit Rev Oncol Hematol 2011 79 315 320 10.1016/j.critrevonc.2010.05.010 20655242

[B15] Hughes KS Schnaper LA Berry D Cirrincione C McCormick B Shank B Wheeler J Champion LA Smith TJ Smith BL Shapiro C Muss HB Winer E Hudis C Wood W Sugarbaker D Henderson IC Norton L Lumpectomy plus tamoxifen with or without irradiation in women 70 years of age or older with early breast cancer N Engl J Med 2004 351 971 977 10.1056/NEJMoa040587 15342805

[B16] Early Breast Cancer Trialists’ Collaborative Group (EBCTCG) Effects of chemotherapy and hormonal therapy for early breast cancer on recurrence and 15-year survival: an overview of the randomised trials Lancet 2005 365 1687 1717 15894097 10.1016/S0140-6736(05)66544-0

[B17] Mandelblaltt J Kreling B Figeuriedo M Feng S What is the impact of shared decision making on treatment and outcomes for older women with breast cancer? J Clin Oncol 2006 24 4908 4913 10.1200/JCO.2006.07.1159 16983102

[B18] Bouchardy C Rapiti E Blagojevic S Vlastos A-T Vlastos G Older female cancer patients: importance, causes, and consequences of undertreatment J Clin Oncol 2007 25 1858 1869 10.1200/JCO.2006.10.4208 17488984

[B19] Giordano SH Hortobagyi GN Kau S-WC Theriault RL Bondy ML Breast cancer treatment guidelines in older women J Clin Oncol 2005 23 783 791 10.1200/JCO.2005.04.175 15681522

[B20] Faivre J Lemmes VEPP Quipourt V Bouvier AM Management and survival of colorectal cancer in the elderly in population based-studies Eur J Cancer 2007 43 2279 2284 10.1016/j.ejca.2007.08.008 17904353

[B21] Sobin LH, Wittekind C International Union Against Cancer (UICC): TNM classification of malignant tumors 1997 5 John Wiley & Sons, Inc, New York, NY

[B22] Ederer F Axtell LM Cutler SJ The relative survival rate: a statistical methodology Natl Cancer Inst Monogr 1961 6 101 121 13889176

[B23] Dickman PW Sloggett A Hills M Hakulinen T Regression models for relative survival Stat Med 2004 23 51 64 10.1002/sim.1597 14695639

[B24] Bastiaannet E Portielje JEA Van de Velde CJH Craen AJM Van der Velde S Kuppen PJK Van der Geest LGM Janssen-Heijnen MLG Dekkers OM Westendorp RGJ Liefers G-J Lack of survival gain for elderly women with breast cancer Oncologist 2011 16 415 423 10.1634/theoncologist.2010-0234 21406470 PMC3228128

[B25] Land LH Dalton SO Jørgensen TL Ewertz M Comorbidity and survival after early breast cancer A review. Crit Rev Oncol Hematol 2011 81 196 205 10.1016/j.critrevonc.2011.03.001 21536452

[B26] Berrino F De Angelis R Sant M Rosso S Lasota MB Coebergh JW Santaquilani M EUROCARE working group Survival for eight major cancers and all cancers combined for European adults diagnosed in 1995-99: results of the EUROCARE-4 study Lancet Oncol 2007 8 773 783 10.1016/S1470-2045(07)70245-0 17714991

[B27] Weggelaar I Aben KK Warlé MC Strobbe LJ Van Spronsen DJ Declined guideline adherence in older breast cancer patients: a population-based study in The Netherlands Breast J 2011 17 239 245 10.1111/j.1524-4741.2011.01074.x 21477166

[B28] Schonberg MA Marcantonio ER Li D Silliman RA Ngo L McCarthy EP Breast cancer among the oldest old: tumor characteristics, treatment choices, and survival J Clin Oncol 2010 28 2038 2045 10.1200/JCO.2009.25.9796 20308658 PMC2860406

[B29] NIH consensus statement: adjuvant therapy for breast cancer http://consensus.nih.gov/2000/2000AdjuvantTherapyBreastCancer114PDF.pdf 11512506

